# Bioavailability and Pharmacokinetics of Anisatin in Mouse Blood by Ultra-Performance Liquid Chromatography-Tandem Mass Spectrometry

**DOI:** 10.1155/2020/8835447

**Published:** 2020-12-23

**Authors:** Xi Bao, Xiajuan Jiang, Jianshe Ma, Xianqin Wang, Quan Zhou

**Affiliations:** ^1^Department of Pharmacy, The First Affiliated Hospital of Wenzhou Medical University, Wenzhou, China; ^2^Analytical and Testing Center, School of Pharmaceutical Sciences, Wenzhou Medical University, Wenzhou, China; ^3^The Laboratory of Clinical Pharmacy, The People's Hospital of Lishui, Lishui, China

## Abstract

**Background:**

Anisatin is a neurotoxic sesquiterpene dilactone wildly found in plants of the family *Illiciaceae*. Due to morphological similarities among *Illiciaceae* fruits, fatal poisonings are frequent.

**Objective:**

This study is aimed at developing a rapid, simple ultra-performance liquid chromatography-tandem mass spectrometry (UPLC-MS/MS) method to determine anisatin's bioavailability in mouse blood and the method's application to pharmacokinetics.

**Methods:**

Blood samples were preprocessed by protein precipitation using acetonitrile. Salicin (internal standard, IS) and anisatin were gradient-eluted by a mobile phase of methanol and water (0.1% formic acid) in a UPLC BEH C18 column. This step involved using an electrospray ionization source of anisatin at a mass-to-charge ratio (m/z) of 327.1 → 127.0 and IS at m/z 285.1 → 122.9 in the negative ion mode with multiple reaction monitoring.

**Results:**

The calibration curve ranged from 1 to 2000 ng/ml (*r* > 0.995), with the method's accuracy ranging from 86.3% to 106.9%. Intraday and interday precision were lower than 14%, and the matrix effect was between 93.9% and 103.3%. The recovery rate was higher than 67.2%.

**Conclusions:**

The developed UPLC-MS/MS method was successfully used for a pharmacokinetic study of oral (1 mg/kg) and intravenous (0.5 mg/kg) administration of anisatin to mice—the absolute bioavailability of anisatin in the mouse blood was 22.6%.

## 1. Introduction

Humanity has faced many grave respiratory infectious disease challenges in this century. Tamiflu (oseltamivir phosphate), the most clinically effective anti-influenza drug, is currently manufactured from shikimic acid, its key precursor [1, 2]. Plant products from the *Illiciaceae* family provide most shikimic acid, but substantial production is found only in few species [3–6]. More than 40 species of *Illiciaceae* are found around the world, many of which are toxic.

Chinese star anise (*Illicium verum* Hook. f., Bajiao) represents a cultivated species with a high content of shikimic acid; it is famous for its use as both spice and medicine [3, 6–8]. The fruits of the genus *Illicium* Mangcao (*I. henryi.* and *I. lanceolatum* A. C. Smith) are morphologically similar and easily confounded [7, 8]. According to its growing demand, the current shikimic acid manufacturing processes, including the tricky extraction of shikimic acid from microorganisms, have made significant advances already but not matured enough to provide for oseltamivir's production [5, 6]. In case of an influenza outbreak, the price increase of Chinese star anise may give unethical suppliers an incentive to adulterate with cheaper counterfeits and increase prices, whereas lowering quality; in this scenario, poisonings are inevitable. A massive outbreak of such poisonings led the European Directorate for Quality Medicines and the US Food and Drug Administration (FDA) to publish guidelines on Chinese star anise and its adulteration [9–12].

People sometimes can discriminate and identify *Illicium* species based on morphology and flavor, but it requires expertise in Botanics, and in many cases, it does not work when the fruits present trace contamination or powder mixed. Thus, chemical analytical methods appear to be the first choice. According to research [12, 13], anisatin is the specific and principal component causing neurotoxicity, with a median lethal dose (LD50) of 1 mg/kg in mice. Extensive studies have shown that anisatin's acute toxic manifestations include emesis and convulsive seizures. Moreover, studies using a metabolomic approach [14, 15] confirmed that anisatin induced convulsive seizures by inhibiting GABA_A_ receptors—the neurotoxic mechanisms involved alterations of neurotransmission and neuromodulation.

Several chemical methods focusing on differences among *Illicium* species are proposed for anisatin detection [16–19]. TLC/HSI analyses are suggested to differ among 2 or more species [16, 19]; however, this approach can not reflect the fruits' overall characteristics and might fail in the face of its partial adulteration. With the rapid development of the GC/LC-MS detection technology, the analysis accuracy in the highly complex chemical field has been dramatically improved. Zhang et al. [20] have developed a sensitive UPLC-MS/MS method for the quantitative determination of intermediate polarity anisatin in biological samples (plasma, urine, and vomit). Each analysis's cycle time was only 5.0 min, and the limits of quantitation were 2 *μ*g/l for plasma samples. According to our knowledge, the best assay has been reported by Schrage and Shen et al. [21, 22], who used DART-orbitrap-MS; the proposed method is more straightforward and less labor intensive compared with other procedures. It allows for an unambiguous distinction between toxic and nontoxic star anise fruits within seconds without any sample pretreatment.

All the numerous current studies on the *Illiciaceae* family are about identifying species or poisoning symptoms; none of the research has ever focused on the pharmacokinetics of treatment time window for patients suffering from acute poisoning. Considering the severity of convulsive seizures induced by anisatin and the scarcity of reports on its pharmacokinetic, it was necessary to establish an analytical method to characterize the pharmacokinetics of this dynamic drug process in mice [23, 24], which were administered with anisatin. To our knowledge, this is the first study on anisatin's pharmacokinetics in mice, and it may help to understand better the pharmacokinetics underlying its toxicity.

## 2. Materials and Methods

### 2.1. Chemicals

Anisatin (purity >98%) was obtained from Sigma (St Quentin Fallavier, France), and salicin (IS; purity >98%) was obtained from Chengdu Munster Biotechnology Co., Ltd. (Chengdu, China). High-performance liquid chromatography (HPLC) grade formic acid, acetonitrile, and methanol were obtained from Merck (Darmstadt, Germany), and ultrapure (type 1) water was obtained through a Milli-Q water system obtained from Millipore Sigma (Burlington, MA, USA).

### 2.2. Instruments and Conditions

An ACQUITY ultra-performance liquid chromatography (UPLC) H-Class system (Waters Corp, Milford, MA, USA) coupled to a XEVO TQS-micro triple quadrupole mass spectrometer and electrospray ionization (ESI) was employed for the analysis of mouse blood samples.

Separation was performed by an UPLC BEH C18 (1.7 *μ*m, 2.1 mm ×50 mm) column at 30°C. The mobile phase was composed of methanol and water (0.1% formic acid) based on a gradient elution with a flow rate of 0.4 ml/min—the gradient elution was as follows: from 0 to 0.2 min, 10% methanol; 0.2 to 1.4 min, 10% to 75% methanol; 1.4 to 2.0 min, 75% methanol; 2.0 to 2.1 min, 75% to 10%, methanol; and 2.1 to 4.5 min, 10% methanol.

**Figure 1:**
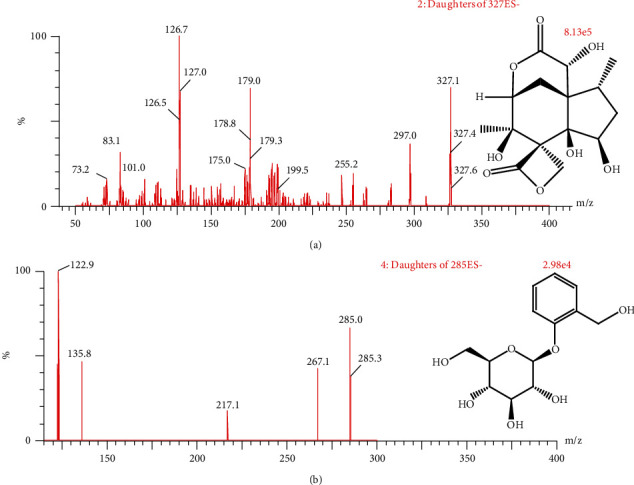
Chemical structure and mass spectrum of anisatin (a) and salicin (IS; b).

The mass analysis was performed under the following conditions: desolvation gas (nitrogen) flow of 900 l/h and capillary voltage of 2 kV; the source of ionization temperature was 150°C, and the desolvation temperature was 450°C. The quantitative analysis was performed in the ESI negative and multiple reaction monitoring mode, obtaining a mass-to-charge ratio (m/z) of 327.1 → 127.0 for anisatin (cone voltage 30 V, collision voltage 12 V) and m/z 285.1 → 122.9 for salicin (cone voltage 30 V, collision voltage 10 V).  Figure 1 depicts the chemical structure and mass spectrum of anisatin and salicin.

### 2.3. Calibration Standards

We prepared anisatin (1.0 mg/ml) and salicin (1.0 mg/ml) standard solutions in methanol and ultrapure water (1 : 1, v/v). Working standard solutions of anisatin were conducted by dilution of stock solutions appropriate with methanol to obtain the desired concentrations of 10, 50, 200, 500, 1000, 2000, 5000, 10.000, and 20.000 ng/ml. Also, the working standard solution of salicin (100 ng/ml) was diluted from its stored standard solution using methanol.

Calibration standards were prepared by spiking blank mouse blood with proper amounts of working standard solutions of anisatin. We constructed a calibration plot for anisatin in blood within the range of 1 to 2000 ng/ml (1, 5, 20, 50, 100, 200, 500, 1000, and 2000 ng/ml), and 3 quality control (QC) samples (2, 180, and 1800 ng/ml) were also prepared in the same way as calibration standards. All the solutions were stored at −20°C.

### 2.4. Preparation of Samples

Blood (20 *μ*L) was added with 100 *μ*L of acetonitrile containing IS at 100 ng/mL. The mixture was vortex mixed for 1 min and centrifuged at 13,000 rpm for 10 min at 4°C. Then, 2 *μ*L supernate was transferred and injected into the UPLC-MS/MS system for analysis.

### 2.5. Method Validation

In accordance with the FDA bioanalytical guidelines, a full validation was performed for the UPLC-MS/MS method [25, 26].

#### 2.5.1. Selectivity

The selectivity of the UPLC-MS/MS method was investigated by comparing peak responses blank mouse blood, blank mouse blood spiked with anisatin and IS, and a mouse sample.

#### 2.5.2. Linearity

A calibration curve (1-2000 ng/ml) for anisatin was obtained in triplicate and generated by plotting the peak area ratio (anisatin/IS; *y*) against the standard nominal concentration (*x*) using 1/*x*.

#### 2.5.3. Precision and Accuracy

Precision and accuracy were evaluated by measuring mouse blood QC samples (2, 180, and 1800 ng/ml) in 6 replicates for 3 consecutive days. Precision was expressed as relative standard deviation. Accuracy was measured between the average value of QC samples and the true value. The actual concentrations determined in QC samples were calculated using the calibration curve obtained on the same day and compared with the nominal concentrations.

#### 2.5.4. Recovery and Matrix Effects

Recoveries were evaluated by comparing the peak areas measured from QC samples to the corresponding standard peak areas. The matrix effects were evaluated by comparing the blank blood's peak areas with the standard solution after sample treatment and the corresponding standard solution's peak areas.

#### 2.5.5. Stability

The stability tests of anisatin in mouse blood were investigated by analyzing replicate QC samples stored under 3 conditions: short-term stability (2 h at room temperature), long-term stability (−20°C, 30 days), and freeze-thaw stability (3 consecutive freeze-thaw cycles for 3 days, −20°C to room temperature).

### 2.6. Pharmacokinetic Study

We kept 12 mice (male, 20-22 g) provided by the Laboratory Animal Center of Wenzhou Medical University (Wenzhou, China) at the Institute of Cancer Research; the animal certificate number was wydw2019-0983. The animals were maintained in a 12 h light/12 h dark cycle at 25°C and 45% to 65% humidity with ad libitum food. The 12 mice were randomly divided into 2 groups (*n* = 6). Anisatin was precisely weighed and completely dissolved in normal saline. A group was given anisatin (1 mg/kg) by the intragastric administration, and another was given anisatin (0.5 mg/kg) by the intravenous administration. This experiment was approved by the Wenzhou Medical University Animal Care Committee. Blood samples (20 *μ*l) were collected at 0.083, 0.5, 1, 1.5, 2, 3, 4, 6, 8, and 12 h from the caudal vein after administration, then stored at −20°C until analysis.

The data determined by UPLC-MS/MS was fitted by the DSA 2.0 (China Pharmaceutical University, China); bioavailability% = 100% × AUC_po_ × D_iv_/(AUC_iv_ × D_po_), where po stands for oral administration, and iv stands for intravenous administration.

## 3. Results

### 3.1. Method Validation

**Figure 2:**
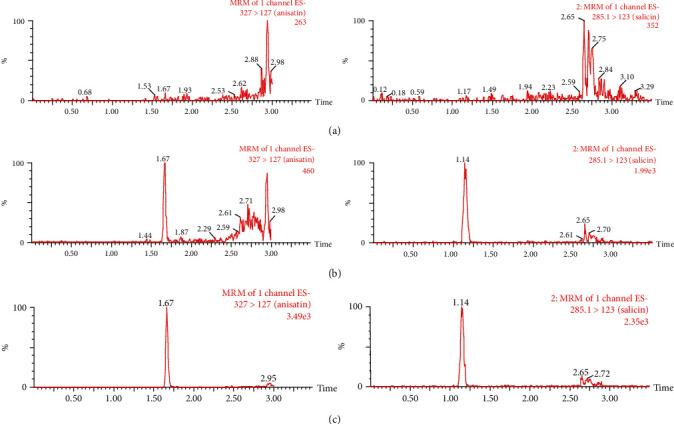
Representative anisatin (*t*_*R*_ = 1.67 min) and IS (*t*_*R*_ = 1.14 min) UPLC/MS/MS chromatograms. (a) A blank blood sample, (b) the blank blood samples spiked with anisatin (0.5 ng/ml) and IS, and (c) blood samples after the oral administration.

Figure 2 shows the representative UPLC-MS/MS chromatogram of a blank blood sample, blank blood spiked with anisatin and IS, and blood sample obtained after the intragastric administration. No potential interference was found in the retention times of anisatin and IS.

The calibration curve equation (1-2000 ng/ml) for anisatin was *y* = 0.0161*x* + 0.0105 (*r* = 0.9987, *n* = 6), *y* represents the ratio of anisatin's peak area to that of IS, and *x* is the concentration of anisatin. The lower limit of quantification (LLOQ) was 1 ng/ml with a signal-to-noise ratio of 10 in mouse blood. The precision and accuracy of the LLOQ were 12.0% and 86.3%, respectively.

Table 1 shows that accuracy ranged from 86.3% to 106.9%, intra- and interday precision were lower than 14%, and a matrix effect between 93.9% and 103.3%, with a recovery rate better than 67.2%.

The stability of anisatin under varied conditions (room temperature for 2 h, −20°C for 30 days, and 3 freeze-thaw cycles) was acceptable; the accuracy was within 85% and 112%, and the precision was lower than 14%.

### 3.2. Pharmacokinetic Study

**Figure 3:**
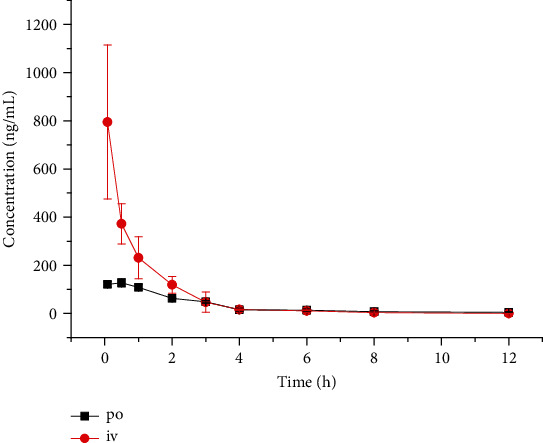
Mean plasma concentration-time profile after the oral (1 mg/kg) and intravenous (0.5 mg/kg) administration of anisatin.

According to the pharmacokinetic results (Table 2), the main pharmacokinetic parameters of anisatin were fitted by the noncompartment model. The blood concentration of anisatin is shown in Figure 3. Bioavailability was 22.6%, which is consistent with good oral absorption.

## 4. Discussion

In mice, the LD50 reported for anisatin ranges from 0.76 to 1 mg/kg (po and ip) [27, 28]; we found the oral toxicity of anisatin standard solutions to be significantly lower than that of the decoction of *I. henryi*—the partial death occurred at 0.5 mg/kg of anisatin from fruit decoction. About 200 kinds of sesquiterpenes have been reported as chemotaxonomic markers of the *Illicium* family, and the toxicity of neoanisatin and 2-oxo-6-deox yneoanisatin has been proved. Toxicity may be enhanced by transformation or synergism between structurally similar sesquiterpenes [29–31].

Mass spectrometry conditions were optimized. We chose the negative mode because the response of anisatin was stronger than that in the positive ion mode; then, fragment peaks with relatively high fragments were selected as quantitative ion pairs, m/z 327.1 → 127.0 for anisatin (cone voltage 30 V, collision voltage 12 V) and m/z 285.1 → 122.9 for IS (cone voltage 30 V, collision voltage 10 V), as shown in Figure 1.

Regarding comparisons between the UPLC BEH C18 column and HSS T3 column, the former proved a better peak shape, and a lower analysis time was obtained with the latter. Different mobile phases were compared, such as methanol, acetonitrile, 10 mmol/l ammonium acetate, and 0.1% formic acid. Methanol and 0.1% formic acid solution in water score especially well in terms of suitable retention time and achieve a better peak.

Choosing a suitable sample treatment method was an essential step in the methodology. The extraction efficiencies of ethyl acetate, acetonitrile, and methanol were compared; acetonitrile extraction efficiencies (around 70%) were better than those of methanol (around 65%) and ethyl acetate (around 55%), with acceptable acetonitrile matrix effects (around 98%).

Selecting the IS was also an important task during the method standardization process. Salicin, astragalin, rubiadin, and narciclasine were compared. Salicin had a better peak shape, a stabler structure, and a peak time similar to anisatin. It was also able to meet the correction function of a IS for this experiment.

The proposed method UPLC-MS/MS is much faster than traditional HPLC for detection of anisatin in mouse blood, more sensitive, and less cost. The total blood volume of rodents is about 7% of their body weight. The number of blood samples, which can be taken from a mouse (20 g), is limited. A mouse has less blood than a rat or rabbit, so it is not easy to get enough plasma volume after centrifuging for liquid-liquid extraction at each time point (a total of 10 time points in 12 h by tail vein transection bleeding). With these factors taken into consideration, only 20 *μ*l of blood samples was collected at each time point by tail vein bleeding, and we chose a one-step protein precipitation procedure for mouse blood. The AUC_(0-__*t*__)_ (area under the plasma concentration-time curve) were 803.6 ± 214.9 and 362.5 ± 16.5 ng/ml × h for intravenous and oral administration, respectively. No quantitative analysis nor pharmacokinetic study of anisatin in mice has been previously reported as far as we know.

## 5. Conclusions

A simple UPLC-MS/MS method was developed to determine anisatin in mice with the LLOQ of 1 ng/ml. The method provides a basis pharmacokinetic for absorption and metabolism of anisatins in mouse blood; the *t*_1/2_ after the oral dose was 5.1 h, and the bioavailability was 22.6%. In addition, the pharmacokinetics of anisatin may provide theoretical support and guidance for the clinical treatment of poisoning episodes.

## Figures and Tables

**Table 1:** The accuracy, precision, matrix effect, and recovery of anisatin in mouse blood samples (*n* = 6).

Concentration(ng/ml)	Precision (%)		Accuracy (RSD^a^%)		Matrix effect (%)	Recovery (%)
	Intraday	Interday	Intraday	Interday		
1	10.6	12.0	94.1	86.3	96.7 ± 7.5	71.9 ± 6.7
2	12.1	13.6	101.8	94.0	93.9 ± 8.2	71.1 ± 5.7
180	8.7	11.6	97.1	95.7	98.8 ± 5.3	75.6 ± 3.2
1800	5.7	8.9	104.1	106.9	103.3 ± 4.2	67.2 ± 3.5

^a^RSD: relative standard deviation.

**Table 2:** Main anisatin pharmacokinetic parameters after oral and intravenous administration.

Parameters	Unit	Po (1 mg/kg)	Iv (0.5 mg/kg)
AUC_(0-__*t*__)_	ng/ml × h	362.5 ± 16.5	803.6 ± 214.9
AUC_(0-∞)_	ng/ml × h	403.6 ± 42.2	804.1 ± 215.2
MRT_(0-__*t*__)_	h	2.5 ± 0.4	1.2 ± 0.1
MRT_(0-∞)_	h	4.3 ± 2.0	1.2 ± 0.1
*t*_1/2*z*_	h	5.1 ± 2.3	1.2 ± 0.1
CL_*z*/*F*_	l/h/kg	2.5 ± 0.2	0.7 ± 0.2
*V*_z/*F*_	l/kg	18.1 ± 7.3	1.1 ± 0.4
*C*_max_	ng/ml	127.3 ± 17.3	795.0 ± 319.8
Bioavailability		22.6%	

## Data Availability

The data used to support the findings of this study are included in the article.

## Abbreviations

ESI: Electrospray ionization
HPLC: High-performance liquid chromatography
IS: Internal standard
LD50: Median lethal dose
LLOQ: Lower limit of quantification
m/z: Mass-to-charge ratio
QC: Quality control
UPLC: Ultra-performance liquid chromatography
UPLC-MS/MS: Ultra-performance liquid chromatography-tandem mass spectrometry.
